# “I’ve Got the Energy to Change, But I Haven’t Got the Energy for This Kinda Therapy”: A Qualitative Analysis of the Motivations Behind Democratic Therapeutic Community Drop-Out for Men With Sexual Convictions

**DOI:** 10.1177/0306624X20956957

**Published:** 2020-09-11

**Authors:** Katie Duncan, Belinda Winder, Nicholas Blagden, Christine Norman

**Affiliations:** 1 Nottingham Trent University, UK

**Keywords:** therapeutic community, sexual offence, attrition, IPA, prison, rehabilitation

## Abstract

Prison-based democratic therapeutic communities (TCs) provide an alternative to mainstream prison, where prisoners can work on psychological difficulties and address offending behavior. Research demonstrates TCs are effective at reducing reoffending rates for residents who stay in therapy 18+ months, and those who drop out of TCs offend at a significantly higher rate than those who complete therapy. Thus, it is important to reduce attrition in TCs. No research has yet explored the explanations for TC drop out offered by those with sexual convictions. The present study uses Interpretive Phenomenological Analysis to qualitatively explore the accounts of men with sexual convictions (*n* = 7) who dropped out of a TC in a UK prison. Results highlight that issues surrounding external responsivity, therapeutic relationships, and treatment readiness were salient in the participants’ accounts of drop out. This research has implications for TCs seeking to better understand and address attrition of people with sexual convictions.

## Introduction

Prison-based democratic therapeutic communities (TCs) are a rehabilitative intervention for prisoners who exhibit elevated levels of psychological disturbance (Shine, 2001). They aim to reduce criminogenic risk and address the psychological symptoms associated with personality disorder (Dolan, 2017). Residents with sexual convictions and those with other crimes, such as violent offences, live together on integrated wings, and the responsibility for decision-making is shared among residents and staff (Akerman, 2019). TCs provide a unique social climate in which every facet of prison life is integral to the process of rehabilitation (Brookes, 2010). Outside of formal therapy groups, the community is an agent of change (Day & Doyle, 2010). Residents monitor and challenge each other’s behavior in a “culture of enquiry” (Shuker, 2013, p. 347), and social learning processes ingrained in interactions between residents and staff create a “living-learning” experience (Kennard, 2004, p. 296). Indeed, Woessner and Schwedler (2014) reported that positive ratings of prison climate were significantly associated with prosocial changes in dynamic risk factors for prisoners residing in a social therapeutic prison, and research has demonstrated that prisoners who complete 18+ months of therapy within TCs demonstrate lower reconviction rates than the general prison population (Cullen, 1994; Miller & Brown, 2010). Based on this evidence, the minimum expected length of stay for many TCs is 18 months; though, official therapy completion could take several years, depending on the individual and their needs (Independent Monitoring Board, 2020).

Birgen (2004) argues that the context in which therapy takes place is an aspect of external responsivity, and this context may be particularly important when it relates to prisoners with sexual convictions, who are seen by the rest of the prison population (and society) as “the lowest of the low” (Griffin & West, 2006). Research has demonstrated that responsivity to therapeutic interventions is bolstered by a climate where prisoners with sexual convictions feel safe (Blagden et al., 2016) and supported by staff (Stasch et al., 2018). Notwithstanding these findings, research by Schmucker and Lösel (2015) demonstrated through a large-scale meta-analysis that treatment programs for sexual offending exhibited significant mean treatment effects only in the community, not in prisons. The evaluation of the previous Sex Offender Treatment Program by Mews et al. (2017) showed no reduction on subsequent sexual recidivism by individuals who had completed the SOTP in prison. These findings suggest the typical prison environment is less conducive to therapeutic change.

However, evidence indicates TCs can be successful in a prison setting (Ross & Auty, 2018). Jensen and Kane (2012) found that completing a TC had a significant effect on reducing the likelihood of rearrest for prisoners. Marshall (1997) conducted a large-scale evaluation of the effectiveness of TCs for people with sexual convictions. In his 4 year follow-up he found that 18% of treated individuals (with two or more previous convictions for sexual offences) were reconvicted compared with 43% of those with sexual convictions who had not received treatment. These findings provide some support for the argument that prison environments with an explicit therapeutic focus are optimal for rehabilitating men with sexual convictions (Akerman, 2010; Blagden et al., 2016; Ware et al., 2010).

Such findings suggest TCs are effective at reducing re-offending; however, a closer consideration of the evidence suggests that TCs are most effective for certain types of resident (Shuker, 2013). Those who complete 18+ months of therapy exhibit significantly lower rates of reconviction compared to residents that leave therapy before 18 months, even after controlling for risk status (Newton & Erikson, 2007). Residents with certain characteristics are reported to remain longer in TCs, especially those who are intelligent (Shine, 2001), low risk (Shuker, 2013) and possess superior psychological insight (Shuker et al., 2007). These results suggest the benefit of TCs is limited to certain residents and, whilst there is a “cautious optimism” (Shuker, 2010, p. 472) regarding TCs’ effectiveness, the results of reconviction studies should not be overstated.

A significant barrier to TC efficacy is high levels of attrition (Rawlings, 1999; Shuker et al., 2007), raising questions about whether appropriate individuals are being selected for therapy and how responsive therapy is to residents’ needs (Newberry & Shuker, 2011). Excessive attrition compromises the cost-efficiency of rehabilitative interventions (McMurran & Ward, 2010) and prisoners who drop out of TCs prematurely waste limited places, leaving others untreated. It is well established that treatment non-completion is associated with higher rates of reconviction over treatment completion (Seager et al., 2004), highlighting concerns about how high rates of attrition may negatively impact public safety (Olver et al., 2011). Another widely cited implication of treatment non-completion is that it increases the risk of re-offending above that of no treatment (Sturgess et al., 2016). McMurran and Theodosi (2007) reported that treatment non-completers were significantly more likely to be reconvicted than an untreated comparison group, suggesting that attending and dropping out of treatment increased individuals’ reoffending risk. However, their meta-analysis included studies that noted differences in pre-treatment risk between the treated and untreated groups, confounding interpretations.

There is a paucity of research into what causes high rates of attrition within prison-based TCs, and the experiences of residents have been largely neglected (Stevens, 2012). Equally, there is a lack of research on the experience of imprisonment for men with sexual convictions (Blagden et al., 2020), which is important as the evidence-base for sex offender treatment programs remains contested (Schmucker & Losel, 2015). Individuals convicted of sexual offences reside at the bottom of the prison hierarchy (McNaughton Nicholls & Webster, 2018) and report feeling victimized and marginalized by other prisoners (Schwaebe, 2005) and staff (Higgins & Ireland, 2009). Ware and Blagden (2016) argue that if we consider what is actually asked of an individual with sexual convictions within treatment, coupled with responsivity factors such as prison environment, then perhaps it is not surprising that the reported sex offender treatment non-completion rates range from 18.9% (Marques et al., 2005) to 80% (Proulx et al., 2004). The threat of violence toward people with sexual convictions in prisons has resulted in them mainly residing on vulnerable prisoner units (VPU), where they are separated from other prisoners (Jewkes et al., 2016). Such prison environments are experienced as anxiety-provoking and hostile, which makes therapy challenging (Ware & Blagden, 2016). However, TCs represent an atypical environment, in which individuals with and without sexual convictions are integrated (Mandikate & Akerman, 2012). The ethos of TCs is to offer an environment with no hierarchy between offences (Stevens, 2012), yet there is limited research that has sought to understand the experience of prison within a TC for men with sexual convictions (Blagden et al., 2019).

The current study aims to add to this limited field of research by qualitatively analyzing the accounts of adult men with sexual convictions who dropped out of a TC in the UK before they had completed therapy. To the authors knowledge there has been no empirical research that has qualitatively considered why people with sexual convictions fail to complete therapy within TCs, despite their experiences being distinctive due to the level of hostility they face from other prisoners (Scrivens & Ricciardelli, 2019). By illuminating the experiences and perceptions of people with sexual convictions who dropped out of therapy before completion, it is hoped this study will bolster knowledge of the issues surrounding TC attrition for this group.

## Method

### Participants

The participants comprised seven adult males convicted of a range of sexual offences who were serving sentences in a category B democratic therapeutic community prison in the UK. Four participants were serving indeterminate sentences for public protection (IPP) and three were serving life sentences. Two participants were housed on a wing for residents with mild learning disabilities. Drop-out included being removed from therapy by staff (*n* = 2) and self-deselection (*n* = 5). The amount of time participants spent at the TC before dropping out ranged from 4 months to 5 years, with a mean of 2.33 years. Whilst most participants stayed longer than the 18-month minimum requirement, they were not deemed to have officially completed therapy as they had outstanding treatment needs to address; and so were considered to have dropped out. The inclusion criteria stated that participants must be in prison for a sexual offence and have dropped out of TC therapy before completion. Participant information is included in Table 1.

**Table 1.** Participant Information.

Participant	Sentence type	Length of stay before drop-out	Mode of drop-out	Resident on a wing for ID
P1	IPP	3.5 years	Self-deselection	No
P2	Life	5 years	Removed by staff	No
P3	IPP	2 years	Self-deselection	Yes
P4	IPP	4 months	Removed by staff	No
P5	Life	3 years	Self-deselection	Yes
P6	Life	7 months	Self-deselection	No
P7	IPP	2 years	Self-deselection	No

### Data Collection

Access was granted to a TC in the UK following ethical approval by HMPPS. The research was advertised using posters located on prison wings. Information sheets were given to research representatives to discuss with other residents during wing meetings. Participants were recruited through the author visiting the wings, talking about the research and arranging interviews directly with interested residents. One-to-one semi-structured interviews were conducted within private rooms in the prison. The interview schedule asked participants about their experiences of the TC and their motivations for dropping out of therapy. Each interview was recorded on a passcode-protected dictaphone and transcribed verbatim. The sample size (*n* = 7) is appropriate for qualitative research utilizing interpretive phenomenological analysis (Pietkiewicz & Smith, 2014).

### Ethics

This research gained ethical approval from HMPPS and Nottingham Trent University. Informed consent was gained from all participants via signed consent forms, which detailed the nature of the study, the limits to confidentiality and the right to withdraw. All participants were provided with contact details of prison staff that could provide support and information. To protect participant confidentiality, participants were given an ID number and any potentially identifiable details were changed in interview transcripts.

### Data Analytical Procedure

This study utilized interpretive phenomenological analysis (IPA) to explore participants’ personal accounts of their motivations behind drop-out from the TC. IPA is an idiographic qualitative approach concerned with making sense of people’s lived experiences and the meanings attributed to those experiences (Smith & Eatough, 2007). The analytical process involves a double hermeneutic; with the researcher “trying to make sense of the participants trying to make sense of their world” (Smith & Osborn, 2003, p. 51). The author read and re-read the interview transcripts to ensure they were fully immersed in the participants’ accounts; a process which facilitates the capturing of emerging themes (Smith & Osborn, 2003). Thought-provoking points within the transcripts were noted in the left-hand margin and recurring patterns of meaning within the descriptive, linguistic, and conceptual coding were developed into subordinate themes. Subordinate themes were then clustered under broader superordinate theme headings.

## Results

Analysis revealed three superordinate themes and their respective sub-themes, presented in Table 2. This study will focus on superordinate themes one and two, as they provided the richest idiographic data. Individual participants will be referred to as P1, P2, etc.

**Table 2.** Superordinate and Subordinate Themes.

Superordinate themes	Subordinate themes
1: (Un)therapeutic climate	1:1 Stigmatized identities and negotiating the “sex offender” label
	1:2 Cracks in the culture
2: Becoming disillusioned with the illusion	2:1 The importance of being authentic
	2:2 Expectations of therapy v reality
3: Experiencing culture shock	3:1 A lack of meaningful information
	3:2 Shedding old values

## Superordinate Theme 1: (Un)therapeutic Climate

This superordinate theme reflects a recurrent narrative within the participants’ accounts that aspects of the wider prison climate acted as a barrier to therapy completion. All participants expressed experiencing stigmatization due to their identity as a “sex offender,” and the integrated environment presented novel challenges for some. Staff not upholding the therapeutic culture within the TC was perceived by participants as creating a double standard, which hampered therapeutic relationships.

### Subordinate Theme 1:1—Stigmatized Identities and Negotiating the “Sex Offender” Label

Participants felt disappointed that their status as an individual with a sexual conviction resulted in them experiencing stigmatization, despite TCs endeavoring to offer an environment with no offence hierarchy (Stevens, 2012). Such perspectives derived primarily from other residents’ directing negative behavior toward the participants.

Extract 1

You know it’s it’s still the same mentality here as a mains prison. You know we’re still sex offenders, and the people still hate us. You know, it’s not equal at all. Erm, I don’t know how you put it, it’s just still that mainstream mentality. I hear it most days, you know, fucking rapist and this that and the other and you know, I just walk by and ignore it all. . .

(P4, lines 303–307)

Extract one highlights the perception that the TC, despite claiming to be different, shares the same mentality as a “mains prison” where “sex offenders” are hated. Repetition of the word “still” conveys the participant’s continued feeling of being labeled, stigmatized, and “not equal” to other prisoners. This sense of othering is echoed by P6, who states “there was people calling people names regarding their offence, saying you shouldn’t speak to them, you shouldn’t speak to them” (lines 77–78). The repetitive experience of not being accepted could increase social isolation, which is elevated within populations convicted of sexual offences (Van den Berg et al., 2018). Increased social isolation is problematic as social isolation is theoretically linked to the etiology and maintenance of sexual offending (Marshall, 2010). Rejection of prisoners with sexual convictions within mainstream prisons is well documented, especially on integrated wings (McNaughton Nicholls & Webster, 2018). However, research has found that in prisons solely for men with sexual convictions, men feel accepted and have the headspace to change, and higher readiness for change (Blagden et al., 2016, 2017). P4 states they “ignore” abuse but describe feeling hated, demonstrating that others’ negative perceptions are internalized. The internalization of negative labels ascribed to those with sexual convictions may result in such individuals developing “self-stigmatizing” attitudes (Mullins & Kirkwood, 2019), which can continue to stifle positive identity transformation even in the absence of externally enacted discrimination (Hildembrand, 2019).

Extract 2

I don’t never wanna come back, I find it really hard. . .imagine being a sex offender around murderers just try an imagine it. Umm well whether I deserve that or whatever umm its bloody hard. . .I was getting sort of bullied in a way but the staff are brilliant I have to be honest they did stop a lot, well they tried to, but it was very difficult. I went through a lot of hell, but it done me good really. Maybe it was good that it engrained, if people could be that horrible, because I always thought (I’ve) been very popular so for people to suddenly be that horrible does engrain the seriousness of what you done.

 (P5, lines 222–231)

Extract two illustrates how others’ negative perceptions can become internalized and how the label “sex offender” can become a lens by which people orientate themselves, influencing their interactions with others. P5 highlights the difficulties he experienced on an integrated wing, stating he “went through a lot of hell” going from being “popular” to being “bullied” due to being labeled a “sex offender.” The implication in extract 2 is that the participant feels the abuse may be deserved or warranted because of their offence. The internalization of negative labels could lead to the golem effect, whereby the negative perceptions of others reaffirm the participants identity as a “sex offender” and hinder identity transformation (Maruna et al., 2009). The phrase “imagine being a sex offender around murderers” denotes fear; a common feeling for those with sexual convictions, who live under the constant threat of violence in prison (Levins, 2014). The importance of perceived safety for rehabilitative efforts is highlighted by Blagden and Perrin (2016), who reported that individuals convicted of sexual offences were more likely to engage in treatment if they perceived the prison environment to be safe.

Extract 3

It’s obviously an assessment wing it’s mixed, so I was kind of like, like do I judge books by its cover and sit next to someone, this is gonna sound bad, who looks like a sex offender? . . .But people on the landing going “oh what prison you come from?” . . .and my defences were up straight away. . .Erm my natural reaction was to say Nottingham. . .

…then they asked how long I was there, and I think they went “oh, that’s a long time to be in Nottingham prison” and I was just like what have I done here? Then obviously it came out and people were like what did you say that for? And I was like well it’s defences isn’t it? I didn’t know how you would react. You know I’ve been on VP wings I aint been on main wings, so I didn’t know how people would react so I was tryna keep myself safe.

(P1, lines 385–401)

In extract three, P1 outlines debating whether to seek out a resident who looked “like a sex offender” upon arrival at the TC. Prisoners with sexual convictions are “outcasts” (Blagden & Pemberton, 2010), who experience high levels of social isolation (Van den Berg et al., 2018). P1 states his “defences were up” when questioned about his previous prison, and he describes lying to try and keep himself safe. Schwaebe (2005) reported that individuals with sexual convictions create survivable niches in integrated prison environments through establishing viable identities and concealing their offences. P1 attempts to establish a viable identity by lying about which prison he transferred from. As his previous prison only housed men with sexual convictions, and revealing this information would expose the nature of his offence, P1 stated he came from a prison which houses men with a range of different offences (HMP Nottingham). This lie protects P1 as it enables him to construct a new identity and avoid the “sex offender” label. P1’s fears about “how people would react” to his offence further illustrates how negotiating stigmatizing labels can shape relational dynamics; with P1 acting defensively due to perceptions about how others will react. Fear of persecution has been cited as a barrier to treatment for those with sexual convictions (Jewkes et al., 2016); however, many individuals who have committed sexual offences complete therapy within TCs. It is possible that individual factors made the experience of stigmatization particularly salient for this sample, increasing their propensity to drop-out. Indeed, individual characteristics have been linked to treatment outcomes, with internal resources such as coping skills, intelligence, and self-control predicting therapy completion in individuals with sexual convictions (Brunner et al., 2019)

### Subordinate Theme 1:2—Cracks in the Culture

Whilst the previous sub-theme focused on the experience of stigmatization within the TC, the current subordinate theme refers to the negative impact of staff not upholding the therapeutic culture. The failure of staff to uphold the values of the TC resulted in participants feeling there was a double standard, which damaged therapeutic relationships and wasted opportunities for pro-social modeling.

Extract 4

I still feel fully invested, I I just wish I felt the staff were fully invested in me being fully invested, do you know what I mean?. . . cause I will say to em, I will be sat in the office and someone will be doing something and I’ll go well why is that happening? And that’s because the staff aint got no bollocks mate and they won’t do what they’re supposed to.

(P2, lines 593–598)

In extract four, P2 states he wishes the “staff were fully invested” in him being “fully invested,” suggesting he felt his commitment was not appreciated by the professionals overseeing therapy. Wilson and McCabe (2002) identified that having a member of staff who encourages and supports a resident through therapy, labeled a “therapeutic champion,” is vital to the change process within a TC. Indeed, staff holding high expectations of prisoners has been demonstrated to facilitate greater self-belief and behavioral change in a phenomenon known as the “Pygmalion effect” (Maruna et al., 2009). P2 states staff lack the nerve to “do what they’re supposed to do,” indicating his doubts surrounding staff competence. Perceptions of staff are pivotal in the rehabilitation of those with sexual convictions, who are typically suspicious of those in positions of power due to histories of betrayal (Serran & Marshall, 2010). Shingler and Mann (2006) argue that working collaboratively and showing a mutual commitment to the therapeutic process is vital to bolster engagement in treatment for those with sexual convictions.

Extract 5

(Staff) they don’t like you challenging them. It’s about you, not about them. Well you’re (staff) part of the community, so you should be up for challenging as much as we are, so I challenged them and they didn’t like it.

(P3, lines 709–712)

Extract five highlights a common narrative throughout the participants’ accounts that there was a perceived double standard with regards to upholding TC values. Whilst residents are expected to challenge each other, P3 states staff were not “up for challenging” and did not respond well to being challenged. This contradiction between staff expectations and behavior is a source of frustration for the participant and may reflect a digression from the regime of pro-social modeling encouraged within TCs (Shuker, 2013). P3’s frustration with staff responding inadequately to challenge may be indicative of a deeper dissatisfaction with the perceived lack of equality between staff and residents. This perception may be especially pertinent as TCs claim to have flattened hierarchies, in which all members have an equal voice (Clarke, 2017). Staff not responding to challenge may indicate to residents’ that their voices are overlooked; creating a power imbalance, which is likely to damage therapeutic relationships (Bacha, 2017).

Extract 6

You’re asking us to be open and honest. Right, you’re supposed to be open and honest yourself. You can’t have it both ways. I struggle with that. And I do struggle with it and when I see staff breaking boundaries, right, I think I’m the only one really in this prison, on this wing that do really challenge.

(P3, lines 739–742)

P3 highlights their discontent with staff expecting residents to be open and honest, whilst not being open and honest themselves. Forging strong therapeutic relationships is an integral part of the change process (Ross et al., 2008), and Dahle (1997) reported that a key predictor of treatment readiness is client trust in the treatment provider. Trust can be developed by establishing a therapeutic relationship based on honesty (Drapeau, 2005) and mutual disclosure (Marshall et al., 2003). This trust is not evident in extract six, with P3 conveying a reluctance to be open with staff who are not open themselves. The phrase “you can’t have it both ways” emphasizes the perception of a one-sided relationship, which is unlikely to empower change (Bennett & Shuker, 2010). Repetition of the word “struggle” suggests a battle in which the participant wants to change but feels he is being held back by others’ negative behavior, including “staff breaking boundaries.” P3’s sense of loneliness in upholding the rules may be indicative of a lack of supportive role models, which would negatively impact the rehabilitative process (Cherry, 2014).

## Superordinate Theme 2: Disillusionment With the Illusion

This superordinate theme reflects a narrative in participants’ accounts that conveyed their disillusionment with the pretense of other residents within therapy. Some participants’ viewed therapy itself as an “illusion,” as it did not match their expectations of what therapy should be. This theme will be unpacked within the two subordinate themes “the importance of being authentic” and “expectations of therapy v reality.”

### Subordinate Theme 2:1—The Importance of Being Authentic

An important theme that emerged across all participants’ narratives was the notion that other residents were faking their way through therapy. Participants frequently drew contrasts between how their peers presented within therapy groups and outside on “the landings” and doubted the authenticity of other residents’ contributions in group meetings. Doubts about others authenticity appeared to stifle participants willingness to be fully open themselves, and an atmosphere of distrust reduced group cohesiveness, resulting in some participants questioning the purpose of engagement.

Extract 7

(residents) pull out because they think that it’s all shit because everyone’s being fake and all that lot. They’ve got their heart and soul and put it into it but they can’t quite get to the point where it means something to them, dya know what I mean? Cause everyone else is being fake, there’s other people coming in and you think why the fuck am I doing this? No one else is fucking doing it.

(P2, lines 741–746)

The common suspicion that everyone else “is being fake” appears to create an atmosphere of distrust within the TC, in which other residents’ motives are questioned and doubted. The need to feel that other residents were being genuine during therapy was salient throughout all participant accounts and appeared to have important implications for their willingness to engage and be open themselves. As therapy within a TC involves divulging deeply personal experiences, it is important that groups create a trusting and safe space for disclosure (Yalom, 1995). Having a safe space in which to share experiences may be an especially pertinent consideration for those convicted of sexual offences, who experience significant levels of shame (Marshall et al., 2009). Shame involves the global evaluation of the self as bad or inferior (Tangney, 1999), intensifying feelings of exposure and vulnerability when under scrutiny (Smith et al., 2002). It is possible that internal feelings of shame presented a barrier to engagement for participants, contributing to their decision to drop out. Residents’ reluctance to put their “heart and soul” into therapy is indicative of a lack of trust, as they do not feel safe to show vulnerability in front of “fake” peers. Not allowing therapy to become meaningful is an emotion regulation strategy that allows residents to retain control and protect themselves from disappointment or rejection (Gross, 2002). Fear of rejection may be especially relevant to those with sexual convictions, for whom making disclosures about sexual transgressions would typically expose them to stigma and moral outrage (Scrivens & Ricciardelli, 2019). P2’s perception that no one else is engaging in therapy suggests a lack of group cohesiveness, which is likely to negatively impact upon treatment outcomes (Beech & Hamilton-Giachritsis, 2005).

Extract 8

Even though, its valuable that people say stuff, a lot of the times I’m thinking are people saying it for effect? Do they really care? Because outside would they? I doubt it very much. If there’s too much doubt then it’s not gonna work for me cause I’m gonna constantly think, or maybe be suspicious. Cause I know it’s just not the way this place does stuff, it’s the way I think as well. And and if that’s constantly going around in my head, it’s not productive anymore.

(P1, lines 131–136)

Extract eight conveys a sense that, for group therapy to be “productive,” it is important that other residents are perceived as being authentic. P1 describes doubting other residents’ motives for helping him, and questions whether they would care outside of prison. The uncertainty about whether other residents would care outside of prison could reflect a lack of self-esteem, which is prevalent among those with sexual convictions (Marshall et al., 2009) and can stifle engagement in treatment (Marshall et al., 2003). Indeed, previous research found that positive feelings arising from therapeutic interactions can be uncomfortable to tolerate and might lead to a lack of trust of other group members or staff within a TC (Akerman & Geraghty, 2016). Whether the lack of authenticity is real or a perception of the participant, constant doubts, and a lack of trust appear to have limited the effectiveness of therapy for P1. Similar difficulties with trusting others are described by P6, who states “it’s very hard for me to trust because I’ve had my past issues through childhood. . .I’m very wary of trusting the prison staff. . .and the rest of the inmates” (lines 888–890). The world view that others cannot be trusted is common in populations with sexual convictions; whose life histories are characterized by dysfunctional early attachments and sexual abuse (McCormack et al., 2002). Indeed, P1 admits some responsibility for his lack of trust in others, acknowledging the way he thinks may be detrimental to therapy.

Extract 9

people on the wings thinking they’re big man, right in the groups their big softies. Right and you see um for what they are. Right, and I think you know summet you’re not like this on the landings. But they talk on the landings. And say nothin in the groups. They’ve got an opinion on the landing, but nothing in the group and nothing on the wing meetings, but they’ve got an opinion on the landing. Erm. . .I think really? Really? You like to do all the talking on the landing but you’ll say nothing on the wing? Nothing on the groups? Yeah. . .it’s not it’s not nice.

(P3, lines 678–684)

Jewkes (2005) highlights a distinction between male prisoners’ private selves and the hyper-masculine public personas they present to survive in a culture of domination. This distinction is apparent in extract 9, which outlines the dichotomy between residents acting the “big man” on the prison landings and being “big softies” in therapeutic groups. Group interventions within prisons can facilitate a temporary move away from traditional displays of hyper-masculinity by providing prisoners with a safe environment in which emotional expression and vulnerability is made possible (Buston, 2018; Karp, 2010). However, P3’s dissatisfaction appears to stem from residents who “talk on the landings” but “say nothin in the groups.” The silence of other residents within the group may represent “masking,” a defensive strategy used by male prisoners to stifle or contain emotions that could convey weakness within a culture of emotional stoicism (Crewe et al., 2013, p. 63). Alternatively, it could indicate other residents’ lack of motivation to participate in therapy. Beech and Hamilton-Giachritsis (2005) reported that high group expressiveness, defined as all members participating emotionally in a group, is a precondition for positive behavioral change in therapy for individuals with sexual convictions, and the non-participation of peers in group settings has been cited as a barrier to treatment completion for this population (Sturgess et al., 2016). It is possible that the non-participation of other group members stifled engagement in therapy for P3, culminating in their decision to drop-out.

Extract 10

I wish I could just stand up and say you know listen you’re not here for the right reasons get out, there’s your bag, go. You know. But you know, it’s it’s it’s just so difficult for me to accept that “well he’s here doing it his way” sort of thing, you know. . .I do, you feel the sense of er when when someone’s discussing or talking about a special er a certain thing that that thats just sort of like effectively as if its reading from a book. You know, (they) have it planned and they’ll just read from it.

(P7, lines 231–238)

In extract 10, P7 questions the sincerity of other residents’ disclosures, comparing them to “reading from a book.” This analogy suggests they perceive other residents as disingenuous, using “planned” scripts to act their way through therapy. P7’s frustration with deceitful group members is illustrated by his yearning to send residents away who are not there “for the right reasons.” Doubts about the motives of other group members appears to pose a distraction from therapy for participant seven. Indeed, Howard et al. (2019) argue that retaining unmotivated prisoners on rehabilitative interventions is likely to negatively influence the effectiveness of treatment for others in their cohort, as therapeutic processes are disrupted and behavioral standards compromised.

### Subordinate Theme 2:2—Expectations of Therapy Versus Reality

There was a signification in several participants’ narratives that therapy within the TC did not match their conceptualizations of what was therapeutic, which led to cognitive dissonance (Festinger, 1957). Several participants felt that therapeutic activities outside of formal groups distracted from “real” therapy, and cynicism surrounding the motivation behind certain tasks conveyed a sense of distrust in the staff implementing therapy. Upon receiving therapy that deviated from their expectations, residents deemed the work “irrelevant”; a conceptualization which likely stifled their engagement.

Extract 11

Whatever it is, ya need backing for it and your supposed to bring it to your group to discuss it you know the reasons as as it’s stated therapeutic er reasons. . .in my view takes time away from me and other people to try and discuss errr offending or whatever. . .

To me that is not therapeutic. You know, it it’s not, but obviously the way I I feel, the way they look at it, because it’s been sort of like dragged out you know. . . we’ve had to go through all this other stuff which in my view is totally, a lot of it’s, totally totally irrelevant. You know and I just cannot, cannot accept and understand you know the *sighs* the wasted time on it.

(P7, lines 147–162)

The opinion that wider therapeutic processes, such as gaining backing for jobs, distracted from “real” therapy emerged in several accounts. Investing residents with the responsibility to make collective decisions about community matters provides them with a sense of personal agency over their daily lives and conveys trust, which is essential for personal change (Bennett & Shuker, 2017). However, P7 perceives democratic discussions to be taking time away from addressing offending. He describes discussions being “dragged out” and time being “wasted” on material he deems “totally irrelevant.” P7 believes that therapy should focus on an individual’s offence, akin to the approach of structured treatment programs (Ward & Stewart, 2003). Therapy within TCs has no formal structure or standardized course content (Bennett & Shuker, 2017), representing a move away from the traditional manualized interventions typically undertaken by individuals with sexual convictions. Not being able to “accept and understand” the reality of therapy within the TC, P7 dropped out to complete “the appropriate courses” elsewhere. This rhetoric could be indicative of a lack of cognitive flexibility as P7 appears unwilling to adapt to the unfamiliar modality of therapy within the TC, which many other residents embrace. According to the Multifactor Offender Readiness Model (MORM) proposed by Ward et al. (2004), deeming therapy irrelevant is an internal cognitive factor that indicates a lack of treatment readiness.

Extract 12

why can’t it just be the work? . . . Why does there have to be stuff like rep jobs and jobs thrown into the mix to test you, I don’t see that as therapeutic, it kind of is challenging my thought process of therapy. So, the idea, and I said in group as well, I know looking in the dictionary for a, you know, a a explanation of the word (therapy) is probably my way of justifying leaving or something. . .in there it says therapy is, you know I dunno, calming. I don’t think it says challenging, but basically it’s not feeling calming anymore.

(P1, lines 195–202)

Tools like “rep jobs” help TCs to create an environment that replicates every-day life outside of prison, enabling residents to develop the skills they will need when they return to society (Akerman, 2019). However, a focus on such jobs was deemed untherapeutic by P1, who believed they were designed to “test” residents. Given that the intended purpose of “rep jobs” is to serve the TC and demonstrate commitment to it, the perception that therapeutic activities were a “test” could be indicative of a lack of trust between P1 and those implementing therapy, whose motivations he doubts. Distrust in authority figures is prevalent in men with sexual convictions, who may have been betrayed by those in positions of power in the past (Akerman, 2010). P1’s perception of being tested suggests he feels under scrutiny and is indicative of shame, which increases awareness of actual or assumed scrutiny from others (Proeve & Howells, 2002). P1 states that the TC was challenging his perception of therapy, causing him to look in the dictionary for a definition to resolve such discord. P1 appears to be experiencing cognitive dissonance (Festinger, 1957), as his experience of therapy goes against his beliefs. To mitigate psychological discomfort, P1 seeks information that supports his conceptualization of therapy and outweighs his conflicting experience. The participant acknowledges that looking in the dictionary may have been an attempt to justify leaving therapy early, as he sought confirmation that what he was undergoing was not actually therapy. Individuals seek to maintain a positive self-concept (Mazar et al., 2008), and undermining the value of the TC could be a mechanism used by residents to mitigate any feelings of failure associated with drop-out; allowing them to move on without damaging their self-esteem.

Extract 13

I came into here with my eyes open thinking you know therapy’s this therapy’s that, then the longer I got here it’s just like it doesn’t seem like it is anymore. It seems like your testing peoples’ reactions. I don’t think that’s therapy in my head. . . Yeah and it’s just therapy’s just starting to feel like it’s jumping through hoops and yeah. . .my minds fighting against that and I don’t think they can do anything, changing my mind. . .

(P1, lines 595–602)

In extract 13, P1 states that the TC is no longer matching their pre-conceptions of therapy. The use of the phrase “jumping through hoops” suggests that therapy feels like a performance, requiring the completion of unnecessary tasks. P1’s cynicism about the purpose of therapeutic activities and the motivations of staff overseeing therapy could reflect a deeper distrust of those in authority. Indeed, Blagden et al. (2017) reported that men with sexual convictions experience a level of ambivalence surrounding their relationships with staff, with doubts about staff authenticity tainting even positive relationships. Suspicion toward staff could be elevated for those convicted of sexual offences, who may experience more punitive attitudes and treatment from staff in mainstream prisons due to the nature of their crimes (Lea et al., 1999). The image of the P1’s mind “fighting” could be reflective of an internal psychological battle surrounding treatment non-completion; in which increasing disillusionment with the modality of therapy eventually outweighed their desire to remain in the TC.

Extract 14

I’ve got the energy to change but I haven’t got the energy for this kinda therapy or the patience or the tolerance.

(P1, lines 611–612)

P1 states that, despite wanting to change, he no longer had the energy, patience or tolerance for the therapy offered. A sense of therapy-induced fatigue was common in all participant accounts, with the sustained toll of therapy and eventually culminating in drop-out for several participants. TCs offer an intensive approach to therapy in an emotionally unstable environment, and residents must possess a high level of commitment to remain until completion (Akerman, 2010). A sense of fatigue may be especially salient in the accounts of this sample due to the relatively long time most participants had remained in therapy before dropping-out. However, the experience of emotional fatigue is typically shared by those who complete therapy, and it may be that individual factors compounded the experience of fatigue for participants in this study.

## Discussion

This study sought to gain a deeper understanding of the motivations behind TC drop-out by qualitatively analyzing the accounts of residents who had dropped out of a TC in the UK before completing therapy. The current study contributes to knowledge surrounding TC attrition by highlighting some of the issues surrounding drop out in a sample of individuals convicted of sexual offences. Analysis revealed that issues surrounding external responsivity, relationships, and internal readiness were prevalent in participants’ narratives.

The current study highlights possible external responsivity issues stemming from stigmatization which is felt even within the social climate of a TC prison that is actively trying to be non-stigmatizing. All participants reported facing stigmatization due to their status as a resident convicted of a sexual offence, which has a number of plausible explanations: that having a sex offence is self-stigmatizing (individuals have internalized society’s fear and loathing of those who commit such a crime), that the staff do not sufficiently challenge or uphold this inclusive environment when it comes to sexual offending, and that practicalities caused by the additional constraints of having prisoners with sexual offences mixed with the general group (e.g., more constraints around visits or photos displayed in cells) may undermine the attempts to de-stigmatize those with sexual convictions. Internalization of others’ negative perceptions may have stifled the process of identity transformation (Maruna, 2001) for several participants, who continued to view themselves as “sex offenders” worthy of mistreatment. It is unsurprising that responsivity is low under these conditions (McMurran & Ward, 2010), and bullying was cited as a motivator behind drop-out in several participants’ accounts. The novelty of integration within the TC caused some participants to experience increased levels of vigilance and anxiety about how other prisoners would respond to their offence. As Jewkes et al. (2016) argue, this level of vigilance is deleterious to therapy as individuals with sexual convictions expend more cognitive resources on survival than rehabilitation. The participants’ experiences sit in contrast to research that demonstrates TCs achieve higher ratings on measures of psychological safety than mainstream prisons (Ministry of Justice, 2009). However, such research includes the ratings of all prisoners and staff, meaning the specific experiences of individuals with sexual convictions may be overshadowed.

Alternatively, the responses to stigmatization exhibited by the participants in this study could have been influenced by internal factors, which may not be shared by those with sexual convictions who complete therapy. Thus, whilst feeling stigmatized was a common experience, it alone is not enough to explain drop-out since it does not take into account those with sexual convictions who do not drop out. Nevertheless, taking steps to reduce the fears and anxieties surrounding integration experienced by those with sexual convictions could reduce attrition within TCs, as increased feelings of safety have been demonstrated to bolster treatment responsivity for people with sexual convictions (Blagden & Wilson, 2020). However, most research in this area focuses on therapeutic environments that only house individuals with sexual convictions, and more research investigating the unique challenges presented by integration would be beneficial (Cornish, 2019). Moreover, it is important to remember that the increased vigilance of prisoners with sexual convictions, and their putative lack of trust in staff at the TC, will be a consequence of their previous experience. Instead of dropping out equating to failure, it may indicate a need for better preparation for individuals pre-arrival, and individuals may need a couple of attempts at starting the TC before they feel able to “complete” therapy. Future research should also consider whether an initial dropout is a necessary stepping-stone for some individuals embarking on a TC, and that more support should be given to such individuals to understand why they dropped out and encourage them, where appropriate, to re-engage. Failure to do so may leave individuals feeling more of a failure, which has potential implications for reoffending.

Analysis highlighted relational issues between participants and staff within the TC. The feeling that staff were not committed to therapy placed a strain on therapeutic relationships, with one participant’s perception that staff were not invested in him contributing to him leaving therapy. Ensuring staff are supportive of therapy is a relevant consideration for TCs seeking to reduce attrition, as residents having a “therapeutic champion” has been demonstrated as vital to the change process within TCs (Wilson & McCabe, 2002, p. 279). Relational issues were also present between participants and other residents. There was a recurrent suspicion that other residents were “faking” their way through therapy, which led some participants to question the purpose of their own engagement and drop-out of the TC. Trust is an important part of forming relationships within a TC that enable residents to feel safe to do the work necessary for change (Dolan, 2017). Group cohesiveness and mutual trust benefits a range of therapeutic processes in rehabilitative interventions utilizing groupwork modalities (Howard et al., 2019), and taking steps to minimize suspicion between residents could be a strategy worth exploring for TCs aiming to reduce attrition. Although distrust could potentially be an issue for any participant in a TC, it is perhaps particularly likely for those with sexual convictions.

The focus in the current findings across several of the themes on the behavior of others in the TC could be a product of feelings of shame. Research indicates that shame motivates the externalization of blame, which acts as a defensive mechanism distracting individuals from distress and helping them regain a sense of superiority (Tangney, 1990). Shame has been reported to negatively impact on therapeutic outcomes if not appropriately addressed (Marshall et al., 2009). Given the discussion above on internalizing the stigma that sexual offending brings it is possible that shame is particularly present in this group, which may have implications for drop out.

Some participants exhibited issues with internal readiness, demonstrated through negative appraisals of the type of therapy offered by the TC. The disparity between participants’ conceptualization of therapy and their experience of therapy in the TC resulted in psychological discomfort. For two participants, this cognitive dissonance (Festinger, 1957) appeared ultimately to be reconciled by dropping out. However, as many residents embrace therapy within TCs, it could be that individual factors reduced these participants’ ability to adapt to the style of therapy within the TC. Nevertheless, negative appraisals of therapy are outlined as a cognitive factor indicative of a lack of treatment readiness within the MORM (Ward et al., 2004), and a substantial body of evidence suggests participants are less likely to engage in treatment they consider irrelevant (McCorkel et al., 1998; McMurran & Mcculloch, 2007; Sturgess et al., 2016). Negative appraisals of the motivations behind therapeutic activities indicated a lack of trust in staff, which is common among those with sexual convictions (Serran & Marshall, 2010). Together with the current qualitative analysis, these results suggests that addressing negative appraisals of therapy and addressing suspicions about staff motives could improve retention of those with sexual convictions within TCs.

It is acknowledged that the findings may highlight the generic experiences of those finding it hard to engage in a therapeutic community, rather than explaining motivations behind drop-out. Some factors perceived to contribute to drop out in this study have also been described in previous research on those who continue within the TC (Akerman & Geraghty, 2016). Importantly some of the strategies (both healthy and unhealthy) for managing difficulties included in such findings are those which might be hard for a person with sexual convictions to engage in. These include having a self-imposed hierarchy in which others crimes are “worse,” feeling safe within the TC and being motivated to continue by family. However, it is still the case that some with sexual offences manage to complete therapy and so further research is needed to elucidate what enables this.

This research represents the accounts of seven participants who were motivated and comfortable to talk about their experience of drop-out with a researcher. A limitation of this research is that the experiences of residents who had dropped out of therapy, but were not interested in participating in the study, were not captured; potentially excluding conflicting accounts. The research is limited by the inclusion of different modes of drop-out within the sample; with some participants being voted out and others self-deselecting. It is possible that the motivations behind these two modes of drop-out are different, and future research should consider separating these groups to determine how mode of exit influences participants’ accounts. Studying those who considered dropping out but did not would be a useful addition to this literature, as would further investigation of what motivates residents to remain in the TC for 18+ months. Future research could explore whether offence type has an impact on prisoners dropping out of therapy. For example, it may be that individuals convicted of sexual offences against children experience the most stigma within TCs and require the most support with responsivity issues.

## Conclusion

This study bolsters psychological understanding of some of the issues surrounding drop out from a TC before therapy completion for residents with sexual convictions. It builds upon the emerging body of evidence surrounding the impact of prison climate on rehabilitative interventions (Akerman et al., 2018), and adds to the limited research area highlighting the perspectives of residents in prison-based TCs. Analysis revealed several issues surrounding external responsivity, internal readiness, and relational issues that were prevalent in participants’ accounts of drop-out. However, these results should be considered alongside possible individual factors that may have heightened the importance of such issues for these participants. Whilst this study does not demonstrate a causal link between participants’ experiences and their decision to drop out, it is hoped these findings will bolster understanding of TC attrition and inform strategies to better retain those convicted of sexual offences within this type of prison environment.
